# Multiomics profiling and experiments in preclinical models revealed RAD51-IN-1 as a synergistic potentiator of anlotinib sensitivity

**DOI:** 10.1126/sciadv.aeb0855

**Published:** 2026-04-15

**Authors:** Huangyang Meng, Qianjing Chang, Yashuang Zhang, Jingjing Ren, Hongyu Guo, Liang Yu, Cheng Qian, Yi Jiang, Lin Zhang, Wenjun Cheng

**Affiliations:** ^1^ Department of Gynecology, the First Affiliated Hospital of Nanjing Medical University, Jiangsu, Nanjing, 210029, China.; ^2^ Maternal and Child Center Laboratory, The First Affiliated Hospital of Nanjing Medical University, Nanjing, China.; ^3^ Affiliated Hospital of Nanjing University of Chinese Medicine and China Traditional Chinese Medicine Obstetrics and Gynecology Reproductive Clinical Medicine Innovation Center, Nanjing 210029, China.

## Abstract

Anlotinib has demonstrated preliminary efficacy as a first-line maintenance therapy for ovarian cancer. However, clinical responses vary widely. To investigate resistance mechanisms and explore rational combinations, pretreatment tumors from 18 patients in clinical trial NCT04807166 underwent whole-exome and RNA sequencing and stratified into sensitive and resistant groups based on progression-free survival. VEGFR-related mutations were enriched in sensitive tumors, whereas resistant tumors showed increased activity of DNA repair pathways and Notch signaling. In vitro screening identified strong synergy between anlotinib and the RAD51 inhibitor RAD51-IN-1, which outperformed the Notch inhibitor FLI-06 in resistant patient-derived organoids. Mechanistic studies revealed that RAD51 inhibition was associated with impaired HRR and increased sensitivity to anlotinib. In vivo, combined treatment with anlotinib and RAD51-IN-1 significantly reduced tumor burden without notable toxicity. These findings suggest that RAD51-mediated HRR may contribute to anlotinib resistance and support RAD51 inhibition as a promising approach to overcome therapeutic resistance in ovarian cancer.

## Teaser

Combining RAD51-IN-1 with anlotinib represents a therapeutic strategy in ovarian cancer.

## INTRODUCTION

Although first-line therapy for ovarian cancer can induce an initial response, high relapse rates remain a major obstacle to long-term survival. Approximately 70% of tumors recur within 3 years of initial treatment (*1*). In recent years, maintenance therapy has been increasingly adopted in the postchemotherapy consolidation phase to delay disease progression. Targeted therapies, particularly antiangiogenic agents such as bevacizumab and poly(ADP-ribose) polymerase (PARP) inhibitors (PARPis) such as olaparib and niraparib, have demonstrated substantial efficacy in patients with breast cancer susceptibility gene (BRCA) mutations or homologous recombination deficiency (HRD) (*2*–*4*). However, the clinical benefits of targeted therapies are limited by considerable interpatient variability and the frequent development of acquired resistance. For example, in the PRIMA (ENGOT-OV26/GOG-3012) trial, 35 to 45% of patients discontinued PARPi treatment because of disease progression or treatment-related toxicity (*5*). Previous studies have identified distinct mechanisms of resistance depending on the type of targeted therapy. For antiangiogenic agents, resistance may arise through the activation of alternative proangiogenic pathways, such as fibroblast growth factor (FGF) signaling. By contrast, for PARPis, resistance is frequently driven by the restoration of DNA repair capacity, which enables tumor cells to overcome HRD and maintain genomic stability (*6*, *7*). Nonetheless, the molecular basis of resistance, particularly to non-PARPis, remains unclear. These limitations underscore the need to identify targeted agents with broader applicability for maintenance therapy in ovarian cancer.

These challenges have prompted interest in multitarget tyrosine kinase inhibitors (TKIs) as alternative maintenance strategies. Among these, anlotinib shows potential due to its broad inhibitory profile. In contrast to PARPis, which primarily benefit BRCA-mutated or HRD-positive patients, anlotinib may offer broader clinical utility. As an orally administered multitarget TKI targeting vascular endothelial growth factor receptor (VEGFR), FGF receptor, platelet-derived growth factor receptor, and c-Kit, its antitumor activity is independent of HRD status. In addition to its broader applicability, anlotinib has demonstrated a more tolerable safety profile during maintenance therapy compared with the hematologic toxicities commonly observed with PARPi. It has demonstrated promising antitumor efficacy across multiple solid tumors, including non–small cell lung cancer (NSCLC) and soft tissue sarcomas (*8*–*11*). Latest findings from the ongoing ALTER-GO-010 study (ClinicalTrials.gov identifier: NCT04807166) demonstrated that first-line treatment with anlotinib combined with carboplatin and paclitaxel, followed by anlotinib maintenance monotherapy, achieved a median progression-free survival (PFS) of 19.58 months in newly diagnosed advanced ovarian cancer (*12*). This compares favorably with historical benchmarks from standard chemotherapy alone (10.3 months in GOG218) and bevacizumab maintenance (14.1 months in GOG218 and 19.3 months in DUO-O) (*13*, *14*). These encouraging outcomes highlight anlotinib as a promising targeted maintenance strategy for ovarian cancer and provide the rationale for our subsequent translational analyses.

Nevertheless, as with other TKIs, treatment outcomes with anlotinib vary among patients, and resistance remains a critical issue (*15*–*17*). Defining the molecular signatures associated with anlotinib sensitivity or resistance and identifying therapeutic vulnerabilities are key to optimizing its clinical efficacy. However, there is a lack of comprehensive studies on the molecular determinants of the response to anlotinib in ovarian cancer, particularly those based on integrative omics approaches using clinical trial specimens.

In this study, we analyzed pretreatment tumor samples from patients enrolled in the trial. Whole-exome sequencing (WES) and transcriptome sequencing [RNA sequencing (RNA-seq)] were performed to identify genomic alterations, transcriptional signatures, and candidate biomarkers associated with the clinical response to anlotinib. Functional validation studies were conducted using in vitro cell lines, organoid models, and in vivo mouse models to elucidate the biological roles of key molecular targets in anlotinib sensitivity and resistance. We also explored potential combination strategies to overcome resistance, with the ultimate goal of informing precision therapy for patients with ovarian cancer. Together, these efforts aim to advance the development of individualized maintenance strategies and improve long-term outcomes for patients with ovarian cancer.

## RESULTS

### Genomic and transcriptomic features of the anlotinib response

**Fig. 1.**
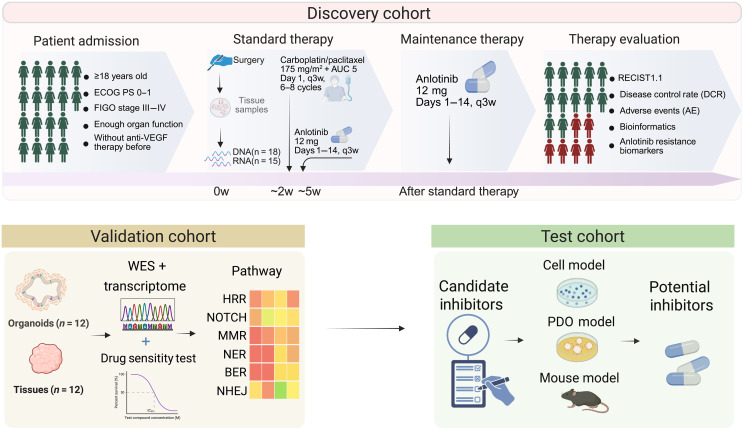
Study design and multicohort strategy for identifying and validating biomarkers of anlotinib resistance in ovarian cancer. Patients meeting the eligibility criteria were enrolled in the discovery cohort and received standard debulking surgery followed by carboplatin/paclitaxel-based chemotherapy and anlotinib maintenance (12 mg, days 1 to 14, every 3 weeks). Eligibility criteria included newly diagnosed FIGO stage III to IV disease, ECOG performance status 0 to 1, and adequate organ function, while patients with prior systemic therapy, uncontrolled comorbidities, or secondary malignancies were excluded. Tumor tissues collected before anlotinib initiation were subjected to WES and RNA-seq to identify candidate resistance biomarkers. Clinical outcomes—including the disease control rate (DCR), adverse events (AEs), and RECIST 1.1 response—were assessed. Candidate biomarkers and pathways (e.g., HRR, NOTCH, MMR, NER, BER, and NHEJ) were further validated in a set of patient-derived organoids (PDOs) and matched tissues (*n* = 12) by drug sensitivity testing and transcriptomic analysis. Potential inhibitors targeting up-regulated pathways were screened in both PDOs and cell lines and validated in vivo in mouse xenograft models to assess their combinatorial efficacy and therapeutic potential. The figure was created in BioRender. H. Meng (2025); https://BioRender.com/18bpic1.

**Fig. 2.**
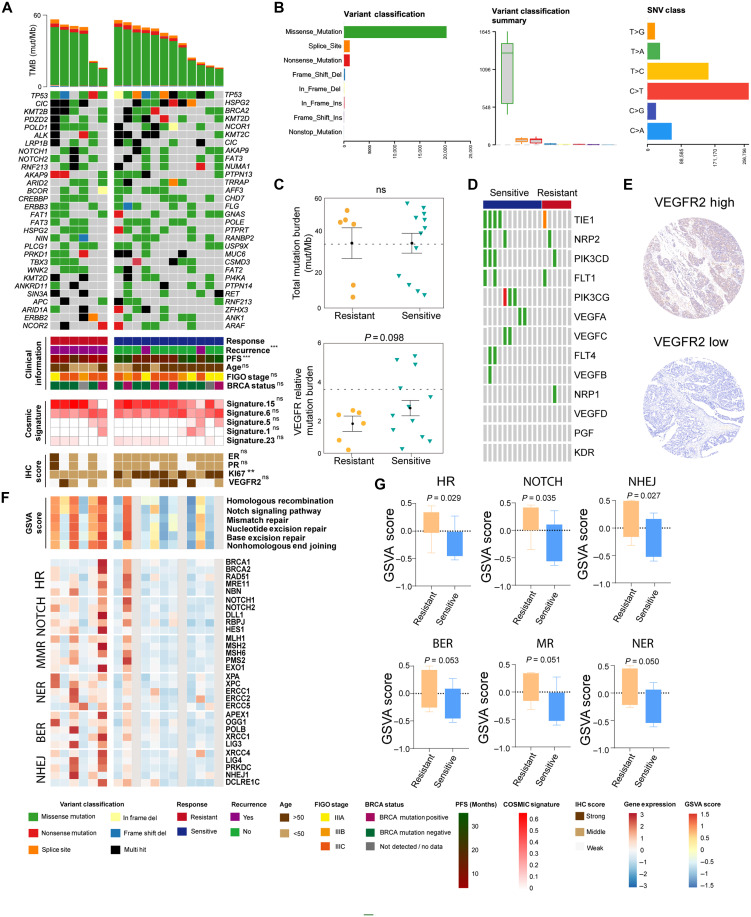
Genomic and transcriptomic differences between anlotinib-sensitive and anlotinib-resistant patients with ovarian cancer. (**A**) Waterfall plot and oncoprint of the top 30 most frequently mutated genes across all patients (*n* = 18). Clinical annotations—including response, BRCA status, cohort group, and key pathway alterations—are shown below. Statistical significance was determined using the Mann-Whitney *U* test for continuous variables and the Fisher’s exact test for categorical data. *P* < 0.05 was considered significant. Significance levels are indicated as **P < 0.01, and ***P < 0.001; “ns,” not significant. (**B**) Distribution of variant classifications and SNV types across the cohort. (**C**) Comparison of total TMB and the VEGFR-related gene mutation load between the resistant and sensitive groups. (**D**) Somatic mutation landscape of angiogenesis-related genes (e.g., VEGFA, FLT1, and NRP1) in sensitive versus resistant tumors. (**E**) Representative immunohistochemistry (IHC) images showing high versus low VEGFR2 expression in tumor tissues; VEGFR2 levels were not significantly different between the groups. (**F**) Heatmap of key genes involved in the DNA repair and Notch signaling pathways. Genes were grouped by functional category: homologous recombination repair (HRR), mismatch repair (MMR), nucleotide/base excision repair (NER/BER), and nonhomologous end joining (NHEJ). (**G**) Gene set variation analysis (GSVA) scores comparing pathway activity between the resistant and sensitive groups. HR (*P* = 0.029), NOTCH (*P* = 0.053), BER (*P* = 0.035), MMR (*P* = 0.051), and NER (*P* = 0.035) pathways were significantly up-regulated in resistant tumors; no significant difference was observed for NHEJ (*P* = 0.27).

A total of 18 patients who met the eligibility criteria were enrolled at our center and received first-line therapy consisting of anlotinib in combination with carboplatin and paclitaxel, followed by anlotinib monotherapy as maintenance treatment. The baseline clinical characteristics of the patients are summarized in Table 1. Tumor tissue samples were collected from all patients before treatment and subjected to WES and transcriptome (RNA-seq) analyses (Fig. 1). Among these patients, 12 achieved a complete response (CR) with a PFS > 12 months and were classified into the anlotinib-sensitive group, whereas the remaining 6 exhibited stable disease or progression with a PFS < 12 months and were categorized into the resistant group. The mean PFS was significantly shorter in the resistant group than in the sensitive group (8.50 months versus 23.08 months, *P* = 0.0021). No significant differences in age, International Federation of Gynecology and Obstetrics (FIGO) stage, or baseline clinical characteristics were observed between the two groups (Fig. 2A).

**Table 1.** Baseline characteristics of patients enrolled in the study. ER, estrogen receptor expression by immunohistochemistry; PR, progesterone receptor expression by immunohistochemistry; Ki67, proliferation index assessed by Ki67 immunostaining. All patients included in this study had high-grade serous ovarian carcinoma (HGSOC). All BRCA mutations reported in this cohort were pathogenic germline mutations.

Patient ID	Age (years)	FIGO stage	BRCA status	Recurrence	PFS (months)	ER	PR	Ki67	Response
P151	69	IIIC	Wild type	Yes	6	Strong	Strong	Moderate	Resistant
P217	57	IIIC	Wild type	Yes	8	Weak	Weak	Moderate	Resistant
P256	44	IIIC	Wild type	Yes	10	Moderate	Moderate	Moderate	Resistant
P58	34	IIIB	–	Yes	11	Weak	Weak	Moderate	Resistant
P106	49	IIIC	Wild type	Yes	4	Moderate	Moderate	Moderate	Resistant
P57	60	IIIC	Mutant	Yes	4	Weak	Weak	Moderate	Resistant
P108	39	IIIC	Wild type	No	31	Moderate	Moderate	Strong	Sensitive
P124	47	IIIB	Mutant	No	30	Moderate	Moderate	Moderate	Sensitive
P354	56	IIIC	Wild type	No	14	Moderate	Moderate	Strong	Sensitive
P147	61	IIIA	Wild type	Yes	14	Moderate	Moderate	Strong	Sensitive
P282	48	IIIC	Mutant	No	15	Moderate	Weak	Strong	Sensitive
P290	55	IIIC	Wild type	No	14	Moderate	Moderate	Strong	Sensitive
P361	52	IIIC	Mutant	No	14	Moderate	Moderate	Moderate	Sensitive
P145	67	IIIB	Wild type	No	29	Weak	Weak	Strong	Sensitive
P66	52	IIIA	Wild type	No	36	Moderate	Weak	Moderate	Sensitive
P143	54	IIIC	Wild type	Yes	18	Moderate	Moderate	Moderate	Sensitive
P152	60	IIIA	–	No	28	Moderate	Moderate	Strong	Sensitive
P1	54	IIIA	Mutant	No	34	Moderate	Moderate	Strong	Sensitive

To investigate the molecular determinants underlying the anlotinib response, we compared the somatic mutational landscape between the two groups. In the entire cohort, missense mutations were the most prevalent variant type, occurring at a much higher frequency than splice site, nonsense, or frameshift mutations. The analysis of single-nucleotide variant (SNV) patterns revealed that C>T transitions were the most common, followed by T>C and C>G substitutions, indicating a preferential bias toward base transitions (Fig. 2B). We next visualized the 30 most frequently mutated genes across the cohort. *TP53*, *CIC*, *KMT2B*, *PDZD2*, and *POLD1* were the most frequently mutated genes. To explore the genomic differences associated with anlotinib response, we compared somatic mutation frequencies between the resistant (*n* = 6) and sensitive (*n* = 12) ovarian cancer samples using Fisher’s exact test (table S1). Several genes exhibited higher mutation frequencies in the resistant group. In particular, *AT-rich interaction domain 2* (*ARID2*) mutations were observed in 50% (3 of 6) of resistant tumors but absent in all sensitive cases (*P* = 0.0245), suggesting potential enrichment in resistant samples. Other genes such as *NOTCH1*, *PRKD1*, *TBX3*, and *WNK2* showed higher mutation rates in the resistant group (each 50% versus 8.3%, *P* = 0.0833), although the differences did not reach statistical significance. *Nuclear receptor corepressor 2* (*NCOR2*) also tended to occur more frequently in resistant cases (33.3% versus 0%, *P* = 0.098). No genes demonstrated significant enrichment in the sensitive group. Overall, these results indicate that mutations in several chromatin remodeling and signaling pathway genes (e.g., *ARID2*, *NCOR2*, and *KMT2B*) may be associated with anlotinib resistance, warranting further validation in larger cohorts (Fig. 2A and table S1). Given that anlotinib is a highly selective inhibitor of VEGFR, we examined the mutational burden of VEGFR-related genes in both groups. Although no significant difference in overall tumor mutational burden (TMB) was observed between the two groups, the sensitive group tended to exhibit a greater mutational load for VEGFR-related genes (*P* = 0.098; Fig. 2C, bottom). Further analysis indicated that VEGFR-related genes including *TIE1* (16.7% versus 33.3%), *NRP2* (16.7% versus 25%), and *PIK3CD* (16.7% versus 25%) occurred at relatively higher frequencies in the sensitive group, whereas *PIK3CG*, *VEGFA*, *VEGFC*, *FLT4*, and *VEGFB* mutations were detected only in a subset of sensitive samples, although no statistically significant differences were observed (Fig. 2D and table S2). These findings suggest that dysregulation of VEGFR signaling may contribute to differential anlotinib responsiveness. However, the immunohistochemistry (IHC) analysis of VEGFR2 protein levels revealed no significant difference between sensitive and resistant tumor tissues, indicating that factors other than VEGFR2 protein expression may mediate anlotinib sensitivity (Fig. 2E). To further explore potential determinants of anlotinib response beyond VEGFR2 expression, we evaluated additional tumor biomarkers commonly associated with ovarian cancer biology. We also compared the expression of estrogen receptor (ER), progesterone receptor (PR), and Ki67 between the two groups. No differences were observed in ER or PR expression, but Ki67 expression was significantly greater in the sensitive group than in the resistant group, suggesting increased proliferative activity. Mutational signature analysis using the Catalog of Somatic Mutations in Cancer (COSMIC) database revealed no significant differences in overall mutational patterns between the sensitive and resistant groups (Fig. 2A, bottom). Collectively, these results highlight that while the overall mutational burden and signatures are similar between the two groups, specific alterations in angiogenesis-related genes and elevated proliferative activity may partially explain the differential response to anlotinib.

To further explore the transcriptional mechanisms of anlotinib sensitivity, we performed gene set variation analysis (GSVA) on the RNA-seq data (Fig. 2F). The resistant group presented significantly elevated activity of multiple DNA repair–related pathways, including homologous recombination repair (HRR), mismatch repair, base excision repair, nucleotide excision repair, nonhomologous end joining (NHEJ), and the Notch signaling pathway (Fig. 2G). Given the link between homologous recombination (HR) activity and BRCA status, tumor WES analysis showed higher BRCA1/2 mutation frequencies in the sensitive group, although without statistical significance. Notably, after excluding BRCA-mutant cases, the sensitive group still exhibited significantly higher HR pathway scores (*P* = 0.0316; fig. S3), indicating that differences in HR activity extend beyond BRCA mutation status. These findings indicate that increased DNA damage repair activity may contribute to reduced sensitivity to anlotinib. Targeting these up-regulated pathways may be a promising strategy to overcome resistance and expand the population of patients who may benefit from VEGFR-targeted therapy.

### Synergistic targeting of DNA repair and notch pathways increased the efficacy of anlotinib in vitro

**Fig. 3.**
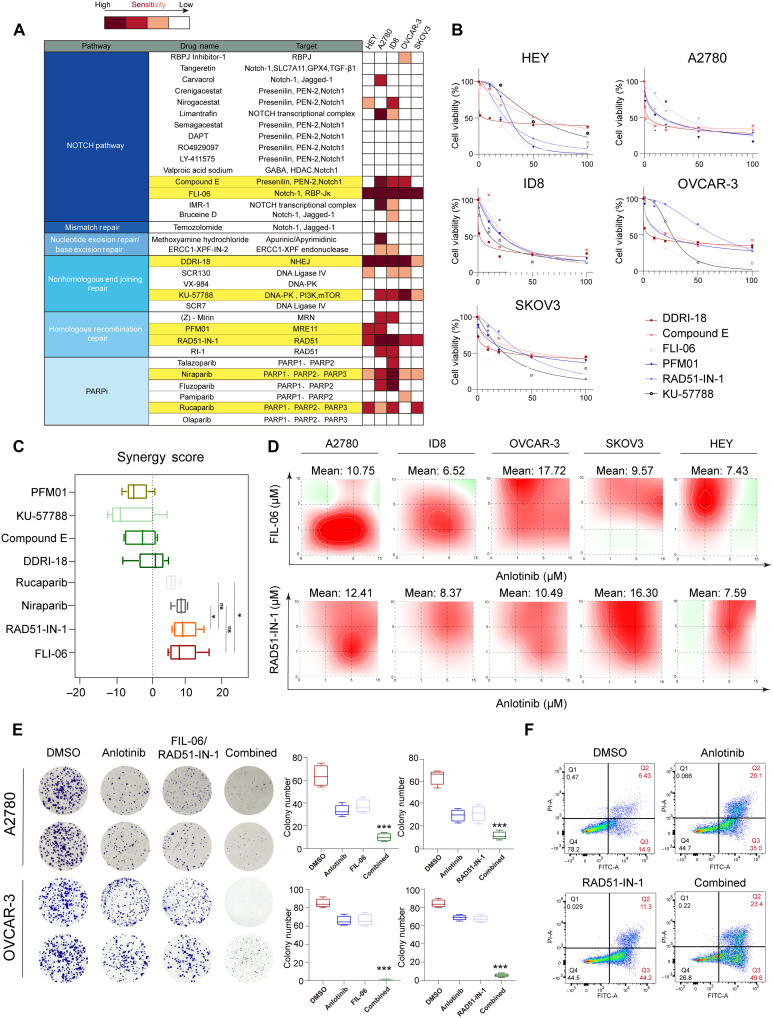
Synergistic effects of pathway inhibitors with anlotinib in ovarian cancer cell lines. (**A**) Summary of selected inhibitors of key resistance-related pathways (NOTCH, HRR, NER/BER, MMR, and NHEJ). Inhibitor sensitivity across five ovarian cancer cell lines (A2780, SKOV3, HEY, ID8, and OVCAR-3) was visualized as a heatmap based on IC_50_ values. (**B**) Dose-response curves showing the effects of representative inhibitors on cell viability across five ovarian cancer cell lines. Inhibitors include DDRI-18, compound E, FLI-06, PFM01, RAD51-IN-1, and KU-57788. (**C**) Boxplots of the synergy scores for eight inhibitors in combination with anlotinib across five cell lines. A synergy score > 10 indicates a synergistic interaction. RAD51-IN-1 and FLI-06 exhibited the greatest synergistic effects on anlotinib. (**D**) Synergy maps showing the combinatorial cytotoxicity of anlotinib and FLI-06 (top) or RAD51-IN-1 (bottom) in five ovarian cancer cell lines. Red indicates the synergistic dose ranges. The mean synergy scores are indicated. (**E**) Colony formation assays of A2780 and OVCAR-3 cells treated with DMSO, anlotinib, FLI-06/RAD51-IN-1 monotherapy, or combination therapy. Right: Quantitative analysis of colony number and area. The combination treatment significantly reduced colony formation. (**F**) Flow cytometry analysis of apoptosis [annexin V–fluorescein isothiocyanate (FITC)/propidium iodide (PI) staining] in A2780 cells subjected to the indicated treatments. Compared with monotherapies, combined treatment markedly increased apoptotic cell death. Significance levels are indicated as *P < 0.05 and ***P < 0.001.

To better understand the determinants of anlotinib response, we first evaluated the sensitivity of multiple ovarian cancer cell lines to anlotinib monotherapy. The median inhibitory concentration (IC_50_) values varied considerably across lines, reflecting intrinsic heterogeneity in drug response. Notably, the BRCA1-mutant, HR-deficient UWB1.289 cell line exhibited the lowest IC_50_, whereas HR-proficient models such as OVCAR-3 and SKOV3 were comparatively less sensitive, suggesting that HRR activity may influence anlotinib sensitivity (fig. S4). To investigate whether targeting hyperactivated pathways in the resistant group could increase the efficacy of anlotinib, we first measured the IC_50_ values of various pathway inhibitors across multiple ovarian cancer cell lines. Considerable heterogeneity was observed both across inhibitors within a single-cell line and across cell lines for the same inhibitor, suggesting that the cellular response to pathway blockade is highly dependent on genetic background. Among the tested compounds, six inhibitors—compound E, FLI-06, DDRI-18, KU-57788, PFM01, and RAD51-IN-1—had relatively low IC_50_ values and minimal intercell line variation, indicating the good stability and broad-spectrum efficacy of these drugs (Fig. 3A and Table 2). Dose-response analyses further confirmed their potent cytotoxicity at low micromolar concentrations (Fig. 3B). Since the ANNIE trial demonstrated the safety and efficacy of combining anlotinib with niraparib in patients with platinum-resistant recurrent ovarian cancer (*18*), we further evaluated the IC_50_ values of available PARPis and assessed their combination indices with anlotinib. Niraparib and rucaparib presented the lowest and most stable IC_50_ values across cell lines and were therefore selected for further synergy analysis (Fig. 3A, bottom).

**Table 2.** IC_50_ values (μM) of candidate compounds across ovarian cancer cell lines.

Compound	A2780	OV3	SKOV3	HEY	ID8
RBPJ inhibitor-1	86.51	33.31	153.1	76.32	42.35
Tangeretin	53.53	70.05	40.77	64.01	85.88
Carvacrol	29.18	45.88	78.26	40.21	50.58
Crenigacestat	169.8	740.6	715.4	156.9	102.7
Nirogacestat	542.7	44.73	46.09	33.01	25.26
Limantrafin	5.236	223.4	882.9	80.96	37.84
Semagacestat	563.0	396.6	345.8	1714.0	252.7
DAPT	212.7	307.2	371.2	162.3	124.5
RO4929097	289.7	130.9	117.3	108.1	116.6
LY-411575	2692.0	98.13	111.0	117.5	79.89
Valproic acid sodium	1.108	148.8	205.8	747.0	251.9
DDRI-18	3.821	5.124	30.3	4.752	1.553
Compound E	0.2304	23.33	56.24	54.25	26.16
FLI-06	15.2	9.946	13.66	19.07	17.95
IMR-1	0.1372	93.95	128.0	85.58	38.63
Temozolomide	6902.0	433.4	304.3	133.5	141.1
Bruceine D	137.5	181.9	85.2	136.1	30.97
Methoxyamine hydrochloride	1.108	193	654.1	4938.0	515.8
ERCC1-XPF-IN-2	44.59	115.6	326.4	94.18	37.75
SCR130	42.72	38.21	44.74	33.34	33.9
VX-984	96.26	47.05	95.92	84.93	64.0
KU-57788	35.34	19.8	39.68	52.84	21.61
SCR7	132.9	163.9	306.6	106.8	87.06
(Z)-Mirin	21.9	127.2	82.51	47.33	23.98
PFM01	16.98	74.62	42.42	28.64	22.35
RAD51-IN-1	15.19	27.61	25.09	24.5	10.78
RI01	22.08	175.6	45.74	40.4	23.27
Talazoparib	172.8	60.8	180.0	80.52	24.78
Niraparib	23.46	33.33	38.56	36.85	13.86
Fluzoparib	21.55	75.3	98.53	88.36	12.96
Pamiparib	248.2	33.49	100.53	127.52	404.7
Rucaparib	36.62	43.08	26.85	21.93	26.95
Olaparib	450.8	>500	162.5	186.36	161.3

The synergy score is a quantitative metric used to evaluate the interaction between drug combinations, helping to determine whether a treatment exhibits synergistic effects and to identify promising therapeutic pairs (*19*). To quantify the combinatorial effects, we calculated synergy scores between anlotinib and each of the eight selected inhibitors. Among these, the HRR inhibitor RAD51-IN-1 and the Notch pathway inhibitor FLI-06—two selective small-molecule compounds that disrupt RAD51 filament formation and Notch receptor trafficking, respectively—showed the strongest synergistic interactions with anlotinib, with synergy scores exceeding those of rucaparib and comparable to those of niraparib (Fig. 3C and fig. S5). Both agents are currently investigational and have not yet been approved for clinical use, but their favorable efficacy and safety profiles in our study suggest potential for future translational development. In five ovarian cancer cell lines, both RAD51-IN-1 and FLI-06 demonstrated strong synergistic cytotoxicity with anlotinib at concentrations less than 10 μM (Fig. 3D). To validate the effects of these combinations on cellular phenotypes, we performed colony formation and apoptosis assays. Both RAD51-IN-1 and FLI-06 significantly suppressed tumor cell proliferation as monotherapies, and their combination with anlotinib further enhanced this inhibitory effect (Fig. 3E). Similarly, each inhibitor alone induced tumor cell apoptosis, and cotreatment with anlotinib markedly increased apoptotic cell death (Fig. 3F and fig. S6). Together, these findings demonstrate that the inhibition of HR or the Notch signaling pathway can significantly increase anlotinib sensitivity in ovarian cancer cells in vitro, supporting the potential of these pathways as combination partners for overcoming anlotinib resistance.

### High RAD51 expression is correlated with anlotinib resistance in PDOs

**Fig. 4.**
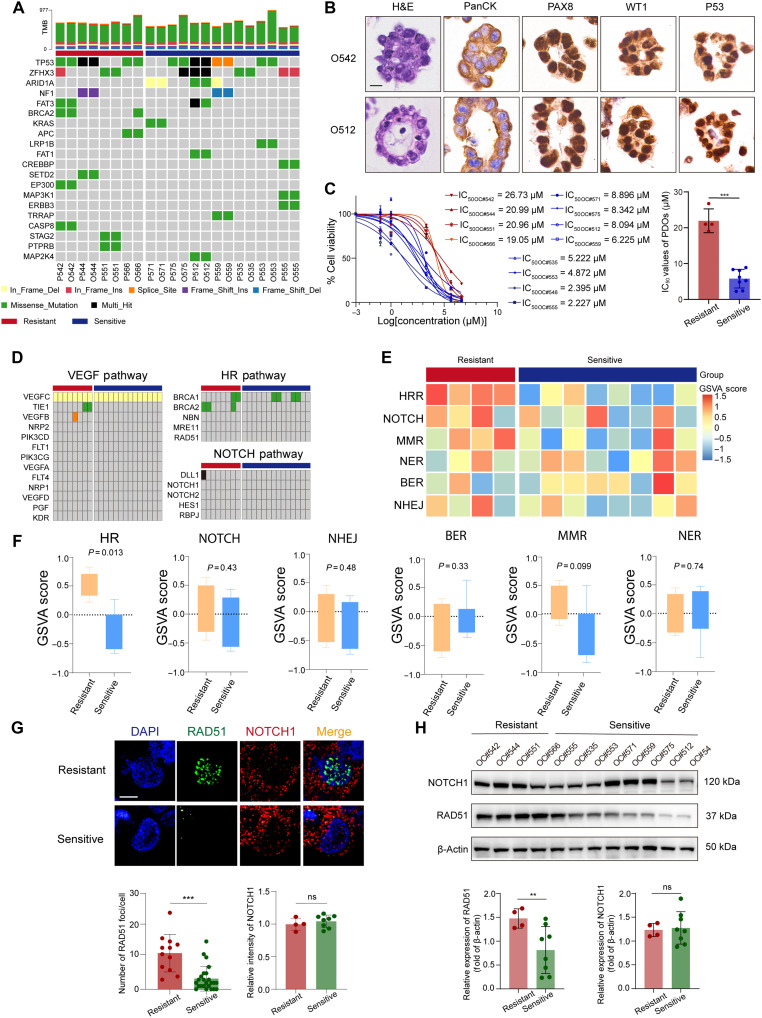
RAD51 overexpression is associated with anlotinib resistance in ovarian cancer PDOs. (**A**) Mutation landscape of 12 PDOs and corresponding primary tumors. Oncoprint showing types and frequencies of mutations in key genes in the sensitive (*n* = 8) and resistant (*n* = 4) groups. (**B**) Morphological and immunophenotypic characterization of representative PDOs (O542 and O512). Organoids retained their epithelial morphology (H&E staining) and expressed the typical high-grade serous ovarian cancer markers PanCK, PAX8, WT1, and P53. (**C**) Dose-response curves and IC_50_ values of anlotinib across 12 PDOs. Resistant PDOs (IC_50_ > 20 μM) are labeled in red, whereas sensitive PDOs (IC_50_ < 10 μM) are labeled in blue. (**D**) Somatic mutation status of VEGF, HR, and Notch pathway genes in resistant versus sensitive PDOs and matched tumors. No consistent difference in mutational burden between groups was observed. (**E**) Heatmap of GSVA pathway enrichment scores for the DNA repair and Notch pathways. (**F**) Comparison of GSVA scores between resistant and sensitive PDOs. Only the HR pathway was significantly more active in the resistant group (*P* = 0.013); the other pathways were not significantly different. (**G**) Representative immunofluorescence images showing RAD51 nuclear foci (green) and NOTCH1 (red) expression in resistant and sensitive PDOs. Images on the left were acquired at ×60 magnification. Scale bar, 10 μm. Quantification of RAD51 foci per cell (left) and relative NOTCH1 fluorescence intensity (right) is presented as means ± SD (*n* = 3 PDOs per group). Statistical significance was determined using an unpaired two-tailed *t* test (***P < 0.001). (**H**) Western blot analysis of RAD51 and NOTCH1 expression in PDO lysates. RAD51 was consistently up-regulated in resistant PDOs (**P < 0.01); NOTCH1 expression was not clearly different.

To further evaluate the preclinical utility of RAD51-IN-1 and FLI-06, we used patient-derived organoid (PDO) model to assess anlotinib sensitivity in ovarian cancer. Genomic profiling by WES demonstrated a high degree of concordance between PDOs and their matched primary tumors in terms of somatic mutations in key oncogenic drivers (Fig. 4A). At the protein level, PDOs retained the expression of canonical epithelial ovarian cancer markers, including PanCK, PAX8, p53, and WT1, confirming the preservation of histological and molecular characteristics (Fig. 4B). Anlotinib sensitivity testing was performed on PDOs derived from 12 patients. Notably, four PDOs presented high IC_50_ values for anlotinib, which significantly exceeded those of the remaining eight organoids (21.93 versus 5.784, *P* < 0.001; Fig. 4C). These four strains were classified into the anlotinib-resistant group, whereas the others were categorized into the sensitive group. Comparative analysis revealed no significant differences in the overall TMB or the somatic mutation load for genes involved in HRR, Notch, or VEGFR signaling pathways between the resistant and sensitive PDOs and their corresponding tumor tissues (Fig. 4D). We next compared transcriptional activity between the two groups (Fig. 4E). GSVA revealed that HRR pathway activity was significantly increased in the resistant PDOs, whereas no meaningful differences were observed in the activity of the Notch, mismatch repair, nucleotide excision repair, base excision repair, or NHEJ pathways (Fig. 4F). At the protein level, Western blot and immunofluorescence analyses revealed significantly greater RAD51 expression in resistant PDOs than in sensitive PDOs, whereas NOTCH1 expression remained unchanged (Fig. 4, G and H). These findings suggest that RAD51 overexpression may be associated with reduced sensitivity to anlotinib, possibly through enhanced HRR activity. In contrast, the up-regulation of the Notch pathway observed at the transcriptomic level may reflect an indirect or downstream response, rather than a primary mechanism of resistance.

### RAD51 inhibition enhances anlotinib sensitivity and is associated with impaired HR repair

**Fig. 5.**
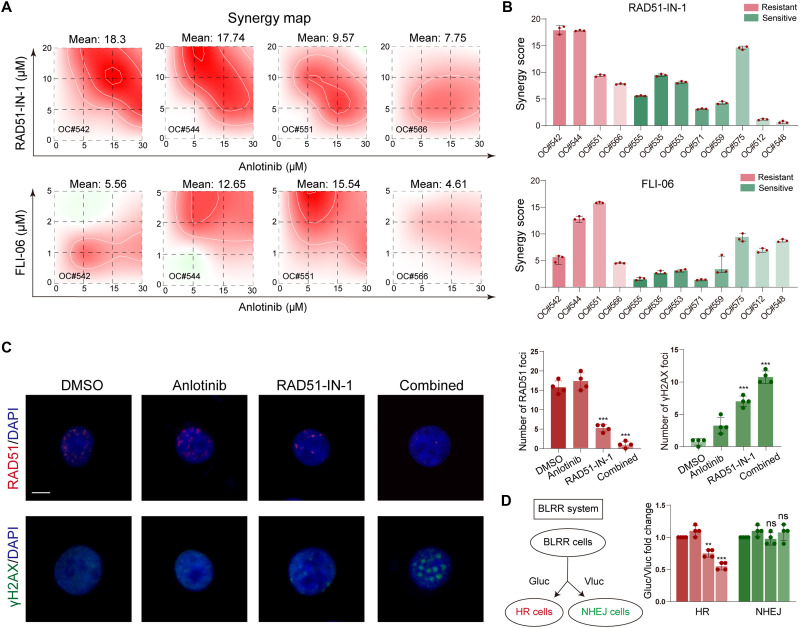
RAD51 inhibition enhances anlotinib sensitivity and is associated with HRR suppression in PDOs. (**A**) Synergy maps showing the combined effect of anlotinib with RAD51-IN-1 (top) and FLI-06 (bottom) in four representative PDOs. Red areas indicate synergy (a greater effect than expected). The mean synergy score is indicated for each PDO. (**B**) Bar graphs showing the synergy scores of RAD51-IN-1 (top) and FLI-06 (bottom) combined with anlotinib across 12 PDOs. RAD51-IN-1 exhibited stronger synergy, especially in resistant PDOs. (**C**) Immunofluorescence analysis of RAD51 and γH2AX foci in OVCAR3 treated with DMSO, anlotinib, RAD51-IN-1, or combination therapy. The number of RAD51 foci was significantly reduced, and the number of γH2AX foci was increased in the combination group. Scale bar, 10 μm. Right: Quantification of RAD51 and γH2AX foci (means ± SD, *n* = 4 cells per group; **P < 0.01 and ***P < 0.001). (**D**) BLRR system used to assess HR and NHEJ repair activity. The schematic shows the Gluc signal indicating HRR and the Vluc signal indicating NHEJ. Combination therapy significantly suppressed HR activity with no change in NHEJ (mean ± SD, n = 3; **P < 0.01, ns = not significant). DAPI, 4′,6-diamidino-2-phenylindole.

To determine whether targeting those two pathways could enhance the efficacy of anlotinib, we assessed the combinatorial effects of RAD51-IN-1 and FLI-06 in PDOs. Both inhibitors demonstrated strong synergy with anlotinib in resistant PDOs; notably, RAD51-IN-1 had a slightly greater combination index than FLI-06. Notably, RAD51-IN-1 also exhibited robust synergy in sensitive PDOs, whereas FLI-06 showed moderate synergy in resistant PDOs (Fig. 5, A and B). These results suggest that inhibition of RAD51 activity may enhance the response to anlotinib, particularly in resistant organoids. To mechanistically validate this effect, we quantified RAD51 and γH2AX foci. Combination treatment markedly reduced the number of RAD51 foci and increased the number of γH2AX foci, indicating impaired HRR activity accompanied by increased DNA damage (Fig. 5C). Furthermore, we used a dual-luciferase reporter system to assess HR and NHEJ activities simultaneously; we found that the combination of RAD51-IN-1 and anlotinib significantly inhibited HR activity without affecting NHEJ (Fig. 5D). Collectively, these findings suggest that concurrent targeting of RAD51 and VEGFR signaling may attenuate HR-mediated DNA repair and enhance cellular susceptibility to anlotinib.

### Combination therapy enhances tumor suppression in vivo

**Fig. 6.**
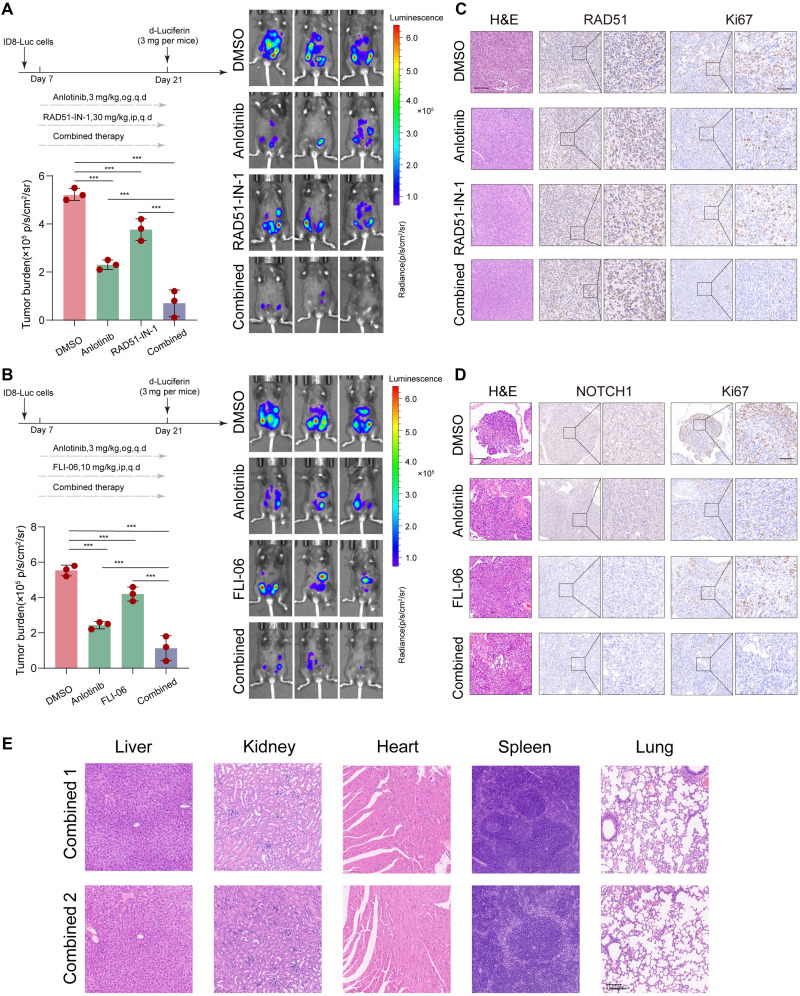
Combination therapy with anlotinib and RAD51-IN-1 or FLI-06 suppresses tumor growth in vivo. (**A**) In vivo efficacy of anlotinib (3 mg/kg) and RAD51-IN-1 (30 mg/kg) monotherapy or combination therapy in ID8-Luc ovarian cancer xenograft mice (*n* = 5 per group). Left: Quantification of the tumor burden by bioluminescence imaging on day 21. Right: Representative luminescence images of each treatment group. Compared with single agent treatment, combined treatment significantly reduced the tumor burden. (**B**) In vivo efficacy of anlotinib (3 mg/kg) and FLI-06 (10 mg/kg) monotherapy or combination therapy in the same model. Compared with monotherapies, combined treatment resulted in enhanced tumor suppression. (**C** and **D**) Representative H&E and IHC staining for RAD51, NOTCH1, and Ki-67 in tumor sections from each treatment group. Images on the left were acquired at ×20 magnification (scale bar, 100 μm) and those on the right at ×40 magnification to illustrate detailed nuclear staining (scale bar, 50 μm). (**E**) H&E staining of major organs (liver, kidney, heart, spleen, and lung) from mice treated with either combination therapy. No significant histological abnormalities were observed, indicating acceptable systemic safety.

To evaluate the therapeutic efficacy and safety of combining anlotinib with either RAD51-IN-1 or FLI-06, we performed in vivo experiments using a murine ovarian cancer model. Bioluminescence imaging revealed a significantly lower tumor burden in the combination treatment groups than in the monotherapy groups (*P* < 0.001), indicating a pronounced antitumor effect (Fig. 6, A and B). Histological analyses using hematoxylin and eosin (H&E) and immunohistochemical staining further confirmed these findings: While RAD51 expression remained largely unchanged, combination treatment markedly suppressed NOTCH1 expression and reduced tumor cell proliferation, as indicated by decreased Ki67 staining (Fig. 6, C and D). This observation may reflect the mechanism of RAD51-IN-1, which inhibits the functional activity of RAD51, rather than its protein expression. Safety assessments revealed no evidence of significant toxicity to major organs, including the liver, kidneys, heart, spleen, and lungs. Histological examination confirmed that the structural integrity of all the organs remained intact (Fig. 6E). Together, these results suggest that the combination of anlotinib with RAD51-IN-1 or FLI-06 confers enhanced antitumor activity with minimal systemic toxicity, supporting its potential for further therapeutic development.

### High RAD51 expression is correlated with anlotinib resistance but not bevacizumab resistance

**Fig. 7.**
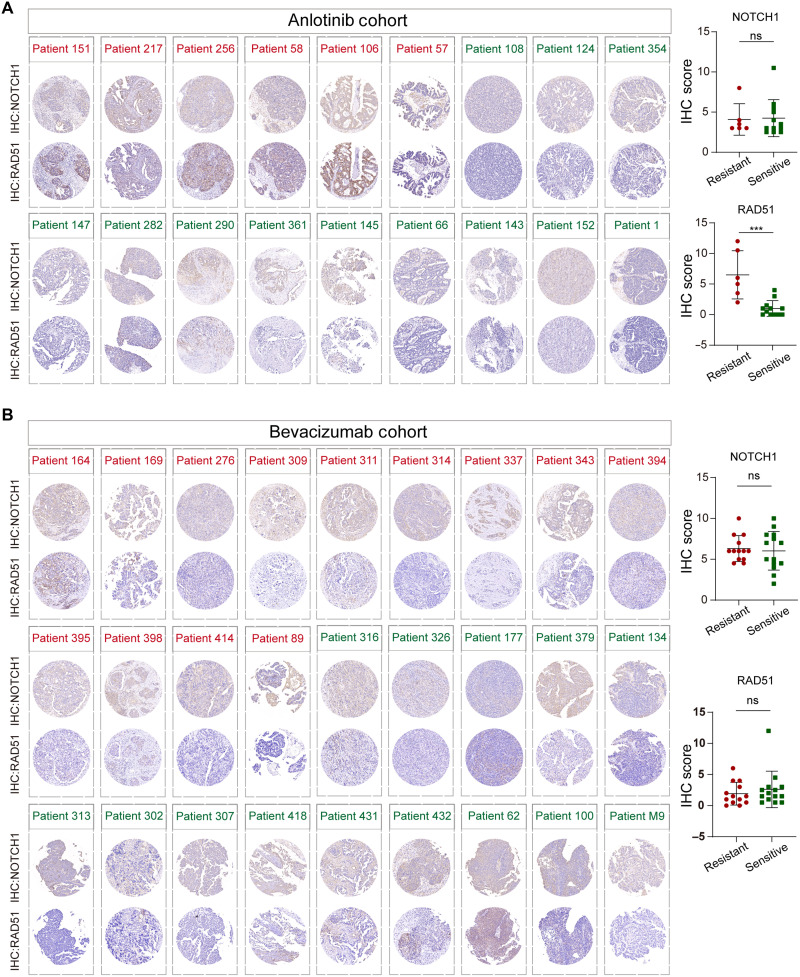
Immunohistochemical analysis of RAD51 and NOTCH1 expression in the anlotinib and bevacizumab treatment cohorts. (**A**) Representative IHC staining of RAD51 and NOTCH1 in pretreatment tumor samples from the anlotinib-treated cohort. Patients were stratified into sensitive (green ID) and resistant (red ID) groups on the basis of PFS. Right: Quantification of the IHC scores revealed that RAD51 expression was significantly greater in the resistant group (***P < 0.001), whereas NOTCH1 expression did not differ significantly. (**B**) IHC staining of RAD51 and NOTCH1 in tumor samples from the bevacizumab-treated cohort, which was stratified in a manner similar to the anlotinib cohort. Right: Neither RAD51 nor NOTCH1 expression significantly differed between resistant and sensitive patients in this cohort.

To further investigate the clinical relevance of RAD51 and NOTCH1 expression in predicting anlotinib sensitivity, we performed IHC analysis of tumor samples from the clinical trial cohort. Consistent with findings in PDOs, RAD51 expression was significantly higher in tumors from the anlotinib-resistant group than in those from the sensitive group (Fig. 7A). In contrast, NOTCH1 expression did not differ significantly between the two groups. Given the mechanistic similarity between anlotinib and the widely used antiangiogenic agent bevacizumab, we also assessed RAD51 and NOTCH1 expression in a separate bevacizumab-treated cohort. The baseline clinical characteristics of the patients in this cohort are summarized in table S3. Based on a PFS cutoff of 12 months, patients were classified as bevacizumab sensitive or resistant. Notably, the expression levels of RAD51 and NOTCH1 did not differ significantly between the two groups in this cohort (Fig. 7B). These findings suggest that the promotion of HRR and aberrant RAD51 expression may be specific features of anlotinib resistance; notably, these features may be mediated by mechanisms other than VEGF inhibition, so further mechanistic investigation is needed.

## DISCUSSION

In recent years, anlotinib has demonstrated promising antitumor activity across various solid tumors (*9*, *11*, *20*, *21*), and its potential as a first-line maintenance therapy in ovarian cancer is gaining increasing attention. However, the emergence of drug resistance remains a major barrier limiting its clinical benefit. The identification of predictive biomarkers and the clarification of resistance mechanisms are therefore critical for expanding the population of patients who may benefit from anlotinib-based therapies. Through a systematic evaluation of patients with ovarian cancer enrolled at our center in the ALTER-GO-010 clinical trial, we observed that, although anlotinib showed substantial therapeutic efficacy in the maintenance setting, clinical responses varied markedly among individuals. To investigate the molecular basis of this variability, we performed integrative genomic and transcriptomic analyses comparing anlotinib-sensitive and anlotinib-resistant patients with ovarian cancer. Our results revealed that elevated activity in DNA damage repair pathways, including HRR, as well as up-regulation of the Notch signaling pathway were key features of resistance. On the basis of these findings, we conducted functional validation using in vitro cell lines, PDOs, and in vivo mouse models. We demonstrated that pharmacologic inhibition of the HRR and Notch pathways markedly increased anlotinib sensitivity, supporting the preclinical therapeutic potential of combination strategies targeting these resistance mechanisms.

Although anlotinib, a multitarget TKI, has been extensively studied for resistance mechanisms in various solid tumors, its mechanism in ovarian cancer is unclear. Previous studies in NSCLC have shown that resistance to anlotinib can be mediated by transcription factor AP-2 alpha (TFAP2A) or exosomal miR-136–5p, which regulate multiple signaling pathways (*22*, *23*). Moreover, anlotinib has been reported to induce protective autophagy in NSCLC cells, and blocking autophagic flux markedly enhances its antitumor activity (*24*). In gastric cancer, cancer-associated fibroblasts promote resistance by suppressing the production of reactive oxygen species induced by anlotinib (*25*). In osteosarcoma, combining anlotinib with tocilizumab (an anti–IL-6R monoclonal antibody) or phosphatidylinositol 3-kinase (PI3K) inhibitors sensitized resistant cells to treatment (*26*, *27*). In contrast, our study is the first investigation on the mechanism of anlotinib resistance in ovarian cancer using prospective clinical trial samples. We identified the activation of HRR and Notch signaling as key features of resistance, and RAD51 overexpression emerged as a potential biomarker. Functional assays confirmed that RAD51 inhibitors, particularly RAD51-IN-1, exhibited robust synergy with anlotinib in resistant cell lines and PDOs comparable to the efficacy of the niraparib. Given the clinical success of combining anlotinib with niraparib in the ANNIE trial (*18*), our findings provide a compelling rationale for the future development of RAD51-targeted combination therapies in HR-proficient ovarian cancer.

Resistance to TKIs involves a complex interplay of mechanisms, including drug metabolism alterations, aberrant PTK pathway activation, evasion of apoptosis, autophagy dysregulation, and adaptations within the tumor microenvironment (*28*). Both RAD51 and NOTCH1, the central molecules in our study, are implicated in multiple resistance-related pathways. For example, tyrosine kinase fusions such as breakpoint cluster region–Abelson murine leukemia viral oncogene homolog (BCR-ABL) fusion gene have been shown to up-regulate RAD51, enhancing DNA double-strand break repair and reducing TKI-induced cytotoxicity (*29*). In addition, Fms-like tyrosine kinase 3 (FLT3) inhibitors such as AC220 impair DNA repair and sensitize FLT3–internal tandem duplication (FLT3–ITD)-positive AML cells to PARP inhibition, illustrating how the DNA repair status influences targeted therapy responses (*30*). Consistent with these findings, we observed that ovarian cancer tissues and organoids with high RAD51 expression were less responsive to anlotinib, while combination treatment with RAD51 inhibitors led to HRR suppression, increased DNA damage, and tumor cell death, suggesting that RAD51 is a key mediator of resistance. However, the predictive utility of RAD51 requires further investigation, as its expression may be influenced by the cell cycle status, DNA damage stress, or upstream regulators such as BRCA2 and CDK12. Future studies incorporating functional assessments, such as RAD51 nuclear focus formation, may provide a more comprehensive evaluation (*31*). Unlike PARPis, which rely on synthetic lethality in HR-deficient cells (*32*), RAD51 inhibitors primarily act by impairing recombinase activity—blocking filament formation on single-stranded DNA and strand invasion—rather than down-regulating protein levels, thereby functionally suppressing HR and offering a complementary rationale for combination strategies. On the other hand, Notch signaling is widely associated with TKI resistance. Several studies have shown that NOTCH1 promotes invasiveness and drug resistance via epithelial-mesenchymal transition (EMT)–related transcription factors such as twist family bHLH transcription factor 1 (TWIST1) (*33*–*35*). In our study, while Notch pathway activity was elevated in the transcriptomic profiles of resistant patients, NOTCH1 protein levels did not differ significantly in either organoids or tumor tissues. This discrepancy may be due to the small sample size or limited coverage of Notch signaling components; thus, further investigation is needed.

We also compared the mutational profiles of sensitive and resistant patients to explore additional mechanisms. TP53 mutations were frequent in both groups, which is consistent with its role in tumorigenesis but suggests limited relevance to the anlotinib response (*36*). Conversely, *RNF213*, *ARID1A*, and *FAT3* mutations were more common in the resistant group, suggesting potential associations with resistance. *ARID1A*, a core component of the SWItch/Sucrose Non-Fermentable (SWI/SNF) chromatin remodeling complex, has been implicated in tumor heterogeneity, immune evasion, and antiangiogenic resistance (*37*). Its loss has been linked to activation of the PI3K/AKT/mTOR pathway, which may counteract VEGFR blockade and undermine the efficacy of anlotinib (*38*). RNF213, an E3 ubiquitin ligase involved in vascular homeostasis and the stress response, may affect microvascular integrity and tumor perfusion (*39*), potentially limiting the antiangiogenic effects of anlotinib. FAT3, a nonclassical cadherin, may promote EMT and drug tolerance through enhanced cellular plasticity (*40*). Notably, mutational types and COSMIC mutational signatures did not differ significantly between groups, suggesting that differences in treatment response are driven more by pathway activity than by specific mutagenic processes. BRCA2 and other HRR core gene mutations were more frequent in the anlotinib-sensitive group, suggesting the presence of a “BRCAness” phenotype, which is known to increase sensitivity to DNA damage-inducing agents such as PARPis (*41*). Furthermore, we observed significantly greater Ki67 expression in the sensitive group than in the resistant group. Although Ki67 is traditionally considered a marker of a poor prognosis (*42*), our findings suggest that highly proliferative tumors may be more dependent on angiogenesis and therefore more responsive to antiangiogenic therapy. These findings suggest that Ki67 may serve as a predictive biomarker for anlotinib efficacy in patients with ovarian cancer; however, further validation is needed in larger cohorts.

Although RAD51-IN-1 remains a preclinical compound, our findings carry important translational implications. RAD51 is essential for maintaining genomic integrity through HRR, and its pharmacologic inhibition represents a rational approach to sensitize tumors to DNA-damaging or antiangiogenic agents. Preclinical studies, including ours, provide proof-of-concept evidence that HRR suppression may enhance the therapeutic efficacy (*43*, *44*). However, clinical translation of RAD51 inhibitors faces challenges, as systemic inhibition could cause toxicity in normal proliferating tissues. Future development will require optimization of selectivity, pharmacokinetics, and safety, as well as tumor-targeted delivery strategies such as nanoparticle or prodrug designs. Identification of biomarkers reflecting HR dependency (e.g., RAD51 foci formation or HRD scores) may also guide patient selection. While RAD51-IN-1 itself may not yet be suitable for clinical use, it serves as a valuable lead compound supporting the feasibility of HRR-targeted combination therapy in ovarian cancer.

Our work should be interpreted in light of several limitations. First, the clinical study was conducted without a comparator arm, meaning that all patients received chemotherapy in combination with anlotinib followed by anlotinib maintenance. Under this design, the molecular signatures we associated with “sensitive” and “resistant” groups could reflect differential responses to the chemotherapy backbone and to anlotinib itself, and we cannot fully disentangle the contributions of each component. Second, BRCA mutations—well-established predictors of platinum sensitivity—may confound the observed associations between HR-related biomarkers and anlotinib response, as improved outcomes in BRCA-mutated tumors could partly reflect enhanced responsiveness to platinum-based chemotherapy rather than to anlotinib per se. However, when BRCA-mutated cases were excluded, the sensitive group still demonstrated higher HR pathway activity, suggesting that broader HRD-related mechanisms may also influence treatment outcomes. Third, the relatively small number of cases included in each group (12 in the sensitive group and 6 in the resistant group) limits statistical power. While statistical testing was performed where appropriate, many of the comparisons remain descriptive, and the associations we report should therefore be regarded as exploratory rather than definitive. Larger, independent cohorts will be required to validate these findings and establish their generalizability. Last, our analyses rely primarily on correlative associations between molecular characteristics and treatment outcomes. Although these patterns are consistent with the hypothesis that HR capacity may modulate responses to anlotinib, direct functional validation—such as manipulating HR genes in isogenic models—will be required to determine whether changes in HR proficiency causally influence drug sensitivity. Nevertheless, we acknowledge the importance of pursuing translational research to identify biomarkers predictive of therapeutic response and resistance. This study represents an incremental but meaningful step toward addressing an unmet clinical need, as predictive biomarkers for anti-angiogenic therapy in ovarian cancer remain scarce.

In conclusion, our study revealed that HRR activation—particularly RAD51 overexpression—is a key mechanism underlying anlotinib resistance in ovarian cancer. RAD51 inhibition markedly enhances anlotinib efficacy, providing a rationale for future clinical trials of individualized combination strategies.

## MATERIALS AND METHODS

### Patient cohort and sample collection

A total of 18 patients with high-grade serous epithelial ovarian cancer were enrolled at our center as part of the ongoing multicenter, single-arm phase 2 trial ALTER-GO-010 (NCT04807166). All patients received first-line carboplatin and paclitaxel in combination with anlotinib, followed by anlotinib monotherapy as maintenance treatment, as outlined in Fig. 1, with baseline clinical characteristics summarized in Table 1. Eligibility criteria included newly diagnosed FIGO stage III to IV disease, ECOG performance status 0 to 1, and adequate organ function, while patients with prior systemic therapy, uncontrolled comorbidities, or secondary malignancies were excluded. Pretreatment tumor tissue samples were collected from all participants for DNA and RNA extraction, followed by WES and transcriptome profiling (RNA-seq). Patients were stratified into two outcome-based groups: The sensitive group was defined as those achieving CR after induction therapy with PFS ≥ 12 months (*n* = 12), while the resistant group was defined as those who did not achieve CR or experienced progression within 12 months (PFS < 12 months; *n* = 6). The 12-month cutoff for PFS was selected on the basis of efficacy end points commonly used in major maintenance therapy clinical trials in ovarian cancer and was further supported by observations from our own cohort. Tumor response was assessed radiologically according to RECIST version 1.1 criteria using computed tomography or magnetic resonance imaging performed at baseline and regular intervals. The cohort presented in Fig. 7B was derived from patients with high-grade serous ovarian cancer treated in our routine clinical practice at the same center, outside of the clinical trial. These patients received first-line paclitaxel plus carboplatin in combination with bevacizumab, followed by bevacizumab monotherapy as maintenance therapy upon completion of chemotherapy. Detailed clinical information for both cohorts is provided in table S3. The study was approved by the Ethics Committee of the First Affiliated Hospital of Nanjing Medical University (approval number: 2021-SR-003), and written informed consent was obtained from all patients.

### WES and somatic mutation analysis

Genomic DNA was extracted from tumor tissues using the FastPure Cell/Tissue DNA Isolation Mini Kit (catalog no. DC102-01, Vazyme, Nanjing, China). Whole-exome libraries were prepared with the Agilent SureSelect Human All Exon V6 Kit and sequenced on the Illumina NovaSeq 6000 platform with paired-end reads. The raw sequencing data were aligned to the human reference genome (hg38) using Burrows–Wheeler Aligner–Maximal Exact Matches (BWA-MEM). Somatic variant calling was performed with Mutect2 from the GATK suite, and variant annotation was carried out using ANNOVAR. The TMB was defined as the number of nonsynonymous somatic mutations per megabase. Mutational signatures and variant spectra were analyzed via the COSMIC database.

### Transcriptome sequencing and pathway enrichment analysis

The total RNA was extracted using the FastPure Cell/Tissue Total RNA Isolation Kit V2 (catalog no. RC112-01, Vazyme, Nanjing, China). The RNA libraries were constructed and sequenced on the Illumina NovaSeq 6000 platform. Gene expression levels were quantified and normalized as transcripts per million values. GSVA was performed on the basis of gene sets from the MSigDB database, including hallmark and Kyoto Encyclopedia of Genes and Genomes (KEGG) gene sets. Pathways of particular interest included HRR, mismatch repair, base excision repair, nucleotide excision repair, and the Notch signaling pathway.

### Cell culture and drug treatment

The human ovarian cancer cell lines A2780 (RRID:CVCL_0134), OVCAR-3 (RRID:CVCL_0465), SKOV3(RRID:CVCL_0532), HEY(RRID:CVCL_0297), and UWB1.289(RRID:CVCL_B079), as well as the mouse ovarian cancer cell line ID8(RRID:CVCL_IU14), were obtained from the Cell Bank of the Chinese Academy of Sciences and authenticated by STR profiling. The cells were cultured in RPMI-1640 or Dulbecco’s modified Eagle’s medium (DMEM) supplemented with 10% fetal bovine serum at 37°C in a humidified incubator with 5% carbon dioxide (CO_2_). Cells were then seeded in 96-well plates at an optimized density of 5 × 10^3^ cells per well and allowed to attach overnight at 37°C in 5% CO_2_. The culture medium was then replaced with fresh medium containing serially diluted drugs prepared in 2% fetal bovine serum medium. For single-agent assays, anlotinib was tested at 0, 1, 5, and 15 μM, corresponding approximately to concentrations around its IC_50_ in the respective cell lines. For combination assays, anlotinib was combined with each candidate small-molecule inhibitor using the same anlotinib concentrations, while the inhibitors were tested at four fixed concentrations corresponding to their individual IC_50_ values (with the highest dose slightly exceeding the single-agent IC_50_). All treatments were performed in duplicate 96-well plates with 0.1% dimethyl sulfoxide (DMSO) as vehicle control. After 48 hours of incubation, cell viability was measured using 3-(4,5-dimethylthiazol-2-yl)-2,5-diphenyltetrazolium bromide (MTT) (Invitrogen) following the manufacturer’s instructions, and background-subtracted values were normalized to the DMSO control. For the two-drug combination analysis, a 4 by 4 matrix was generated for each pair of compounds (16 dose combinations in total). The experiments were performed at least three independent times, and combination indices were calculated using ZIP synergy model in SynergyFinder 3.0.

### Colony formation and apoptosis assays

For the colony formation assays, 500 to 1000 cells were seeded per well in six-well plates and treated with the indicated drugs. After 10 to 14 days of incubation, the colonies were fixed with 4% paraformaldehyde, stained with crystal violet, and manually counted. Apoptosis was assessed by annexin V–fluorescein isothiocyanate and propidium iodide double staining, followed by flow cytometry (BD FACSCanto II). Flow cytometry data were analyzed using FlowJo software (v10.8.1, BD Biosciences).

### PDO culture and drug sensitivity assay

Tumor cells were isolated from fresh tumor specimens of 12 patients with ovarian cancer and embedded in Matrigel to establish PDOs. The methodology for generating PDOs was adapted from the protocol reported by Kopper *et al.* (*45*). Fresh ovarian cancer tissues obtained at surgery were promptly transferred to a sterile biosafety cabinet for organoid construction. Samples were thoroughly washed with precooled PBS, and necrotic areas or blood residues were removed using sterile scissors and forceps. The tissues were cut into approximately 2 mm–by–3 mm fragments and centrifuged. The pellet was resuspended in a digestion solution containing collagenase (SCR103, Sigma-Aldrich, MA) and dispase (D-4693, Sigma-Aldrich, MA) and incubated in a 37°C water bath until dissociation into clusters of 10 to 20 cells. Undigested fragments were filtered through a 70-μm strainer, and the resulting clusters were collected by centrifugation. The cell pellet was then resuspended in Matrigel and seeded as domes in 48-well plates. After polymerization of the Matrigel at 37°C for 20 min, 200 μl of organoid culture medium was added per well. Organoids were maintained at 37°C in 5% CO_2_, with medium changes every 3 days, and passaged every 2 to 3 weeks according to established protocols. Organoids were cultured in customized medium supplemented with defined growth factors to support epithelial ovarian cancer organoid expansion. The phenotypic identity of the PDOs was validated by immunofluorescence and Western blot analyses of epithelial ovarian cancer markers, including PanCK, PAX8, WT1, and P53. PDOs were first dissociated into single cells by enzymatic digestion (collagenase/dispase), followed by gentle pipetting. Cells were seeded into 96-well plates embedded in Matrigel at a density of 100 cells per well and cultured in complete organoid medium for ~7 days to allow reformation of uniform spheroids. Drug testing was initiated when the spheroids entered the logarithmic growth phase (fig. S1). Organoids were treated with a range of anlotinib concentrations, either alone or in combination with candidate pathway inhibitors. Drug exposure lasted for 48 hours, after which cell viability was assessed using the CellTiter-Glo 3D viability assay (Promega). Dose-response curves were fitted using a four-parameter logistic regression model (GraphPad Prism), and IC_50_ values were calculated. PDOs were ranked by IC_50_ values and stratified into tertiles: sensitive (lowest 8), intermediate (middle 8), and resistant (highest 8). Because of the limited specimen size, only four resistant PDOs yielded sufficient material for drug testing and sequencing. Thus, downstream analyses included 8 sensitive PDOs and 4 resistant PDOs, while intermediates were excluded. The full IC_50_ distribution is shown in fig. S2.

### BLRR system assay

To evaluate the activity of the HR and NHEJ pathways, a dual-luciferase reporter system was constructed and used to transfect cells, followed by drug treatment as previously described (*46*). The system-associated plasmids were purchased from Addgene (catalog mo. 158958). pLenti–bioluminescent repair reporter (BLRR) was a gift from C. P. Lai (Addgene, plasmid #158958). Luciferase activities were measured using the Dual-Luciferase Reporter Assay System (Promega) according to the manufacturer’s instructions. HR and NHEJ repair efficiencies were quantified on the basis of normalized luminescence intensity.

### In vivo tumor model and animal experiments

Female C57BL/6 mice (4 to 6 weeks old) were intraperitoneally injected with 5 × 10^6^ ID8 murine ovarian cancer cells to establish a peritoneal tumor model. One week postinjection, the mice were randomly assigned to receive vehicle control, anlotinib (3 mg/kg), RAD51-IN-1 (30 mg/kg), FLI-06 (10 mg/kg), or combination treatment. At the study end point, the mice were euthanized, and tumor tissues and major organs were collected for histological evaluation. H&E staining and IHC were performed to assess the expression of Ki67, RAD51, and NOTCH1. All animal experiments were carried out in accordance with the approved plan of the Institutional Animal Care Committee (IACUC) of the First Affiliated Hospital of Nanjing Medical University (IACUC-2507027).

### IHC, immunofluorescence, and protein expression analysis

Formalin-fixed paraffin-embedded tissue sections were subjected to standard IHC protocols to detect RAD51, NOTCH1, VEGFR2, and Ki67 expression. Immunohistochemical staining was semi-quantitatively assessed using the immunoreactive score (IRS) system, which combines staining intensity (0 to 3) and the proportion of positive tumor cells (0 to 4). IRS was calculated as the product of these two parameters, yielding a final score ranging from 0 to 12. For each case, at least five high-power fields (400×) were evaluated. Two pathologists independently scored the slides in a blinded manner, and the mean score was recorded; therefore, fractional values may appear. For PDOs, protein expression was evaluated by Western blotting and IHC. The primary antibodies used were as follows: anti-RAD51 (1:200 for IHC/IF and 1:20,000 for Western; rabbit monoclonal, Abcam, catalog no. ab133534; RRID:AB_2722613), anti-NOTCH1 (1:100 for IHC and 1:1000 for Western; rabbit monoclonal, Abcam, catalog no. ab52627; RRID:AB_881725), anti-γH2A.X (1:200 for IF; rabbit monoclonal, Abcam, catalog no. ab81299; RRID:AB_1640564), anti-VEGFR2 (1:200 for IHC; rabbit polyclonal, Proteintech, catalog no. 26415-1-AP; RRID:AB_2756527), anti-Ki67 (1:8000 for IHC; rabbit polyclonal, Proteintech, catalog no. 27309-1-AP; RRID:AB_2756525), anti-PAX8 (1:1600 for IHC; rabbit polyclonal, Proteintech, catalog no. 10336-1-AP; RRID:AB_2236705), anti-WT1 (1:200 for IHC; rabbit monoclonal, Abclonal, catalog no. A2446; RRID:AB_3674524), anti-P53 (1:500 for IHC; rabbit polyclonal, Proteintech, catalog no. 10442-1-AP; RRID:AB_2206609), and anti-PANCK (1:2000 for IHC; rabbit polyclonal, Proteintech, catalog no. 26411-1-AP; RRID:AB_2880505). Immunoreactivity was visualized via the use of horseradish peroxidase–conjugated secondary antibodies and 3,3′-diaminobenzidine (DAB) substrate for IHC. For immunoblotting, the signal intensity was quantified using ImageJ software.

### Statistical analysis

All statistical analyses were performed using GraphPad Prism 9 (GraphPad Software, San Diego, CA, USA) and R software (version 4.3.1). Continuous variables were compared using Student’s *t* test or the Mann-Whitney *U* test, as appropriate. Categorical variables, including gene mutation frequencies and clinicopathologic characteristics, were compared using the Chi-square test or Fisher’s exact test when expected counts were <5. Correlations between gene expression levels and clinical parameters were assessed using Spearman’s rank correlation. A synergy score greater than 10 was considered indicative of synergistic interaction in combination treatment assays. All *P* values were two-sided, and *P* < 0.05 was considered statistically significant.
