# Supplementary material for: Multiomics profiling and experiments in preclinical models revealed RAD51-IN-1 as a synergistic potentiator of anlotinib sensitivity
Source: Sci Adv. 2026 Apr 15;12(16):eaeb0855. doi: 10.1126/sciadv.aeb0855 (PMC13082322; doi:10.1126/sciadv.aeb0855)
Supplement: Supplementary file 1 — Figs. S1 to S7 Tables S1 to S3 Legend for data S1 [file sciadv.aeb0855_sm.pdf]

Science Advances
AAAS

Supplementary Materials for

Multiomics profiling and experiments in preclinical models revealed RAD51-IN-1 as a synergistic potentiator of anlotinib sensitivity

Huangyang Meng *et al.*

Corresponding author: Lin Zhang, davidzhang@njmu.edu.cn; Wenjun Cheng, chengwenjun@jsph.org.cn

Sci. Adv. **12**, eaeb0855 (2026)
DOI: 10.1126/sciadv.aeb0855

The PDF file includes:

Figs. S1 to S7
Tables S1 to S3
Legend for data S1

Other Supplementary Material for this manuscript includes the following:

Data S1

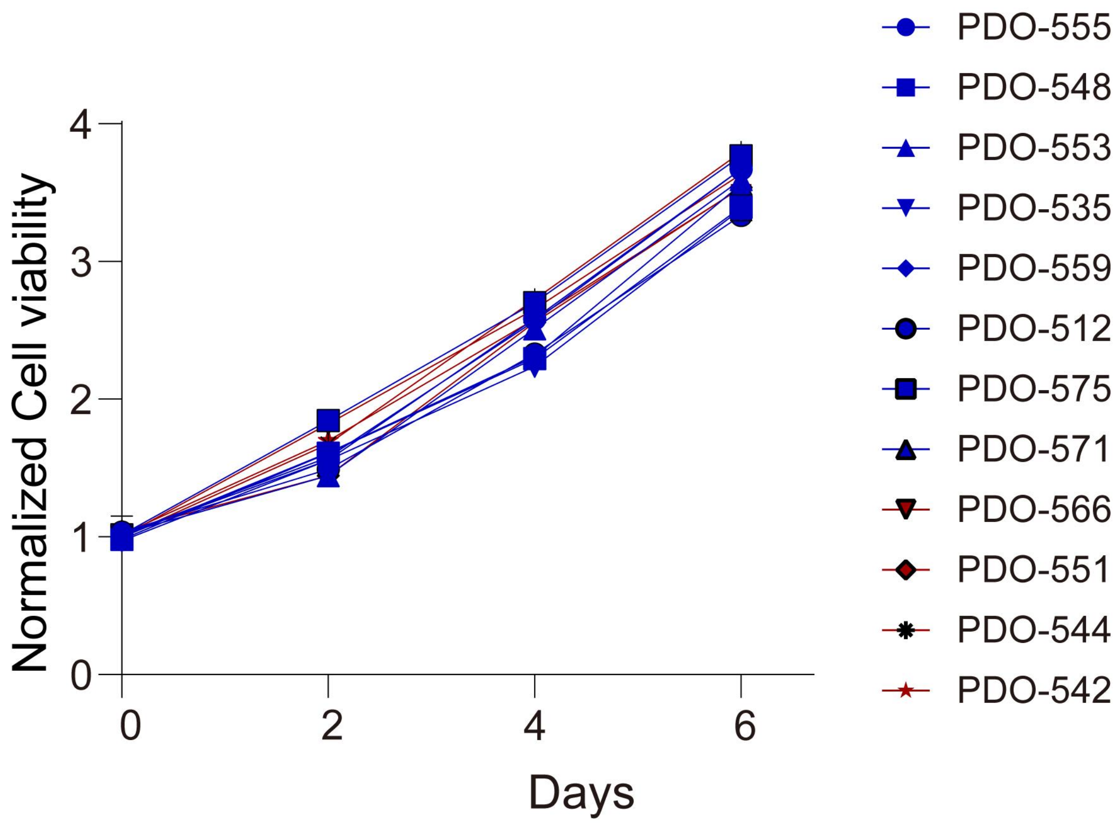

Figure s1. Growth curves of 12 patient-derived ovarian cancer organoids (PDOs) under standard culture conditions. All PDOs demonstrated comparable proliferation kinetics, indicating consistent growth behavior under standardized conditions. Y-axis represents normalized cell viability (fold change relative to Day 0).

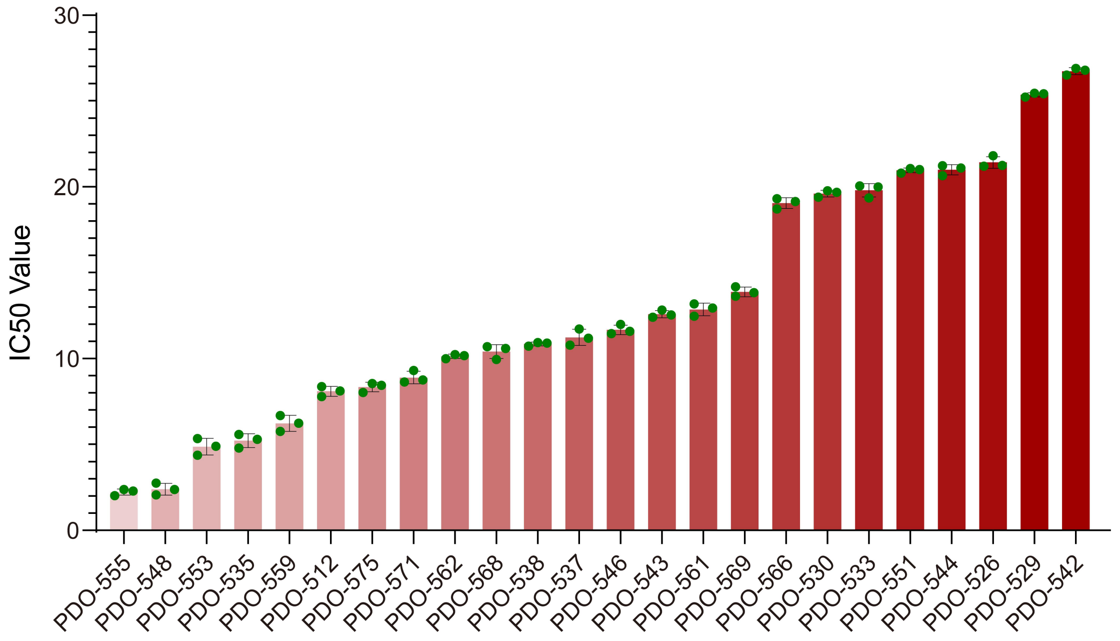

Figure s2. Distribution of IC50 values across 24 patient-derived ovarian cancer organoids (PDOs) following anlotinib treatment. Each bar represents the mean IC50 value for an individual PDO, with biological replicates indicated by green dots.

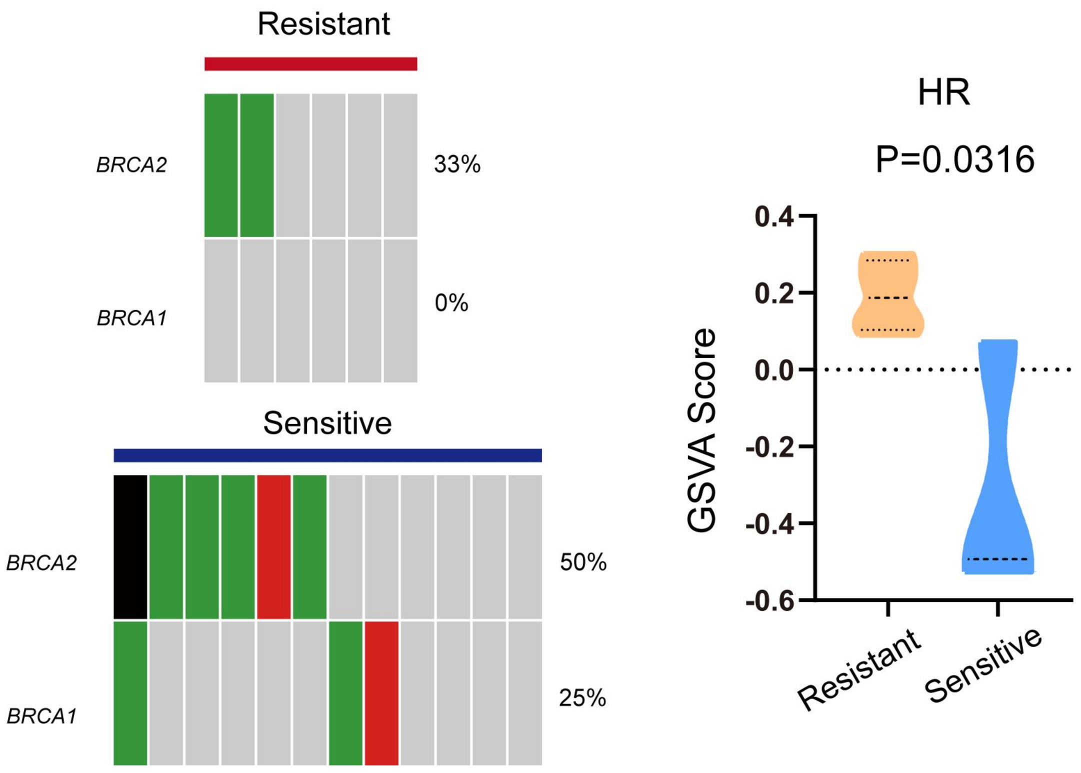

Figure s3. BRCA mutation status and homologous recombination (HR) pathway activity in anlotinib-sensitive and -resistant ovarian cancer samples. (left) Distribution of BRCA1 and BRCA2 mutations in the resistant and sensitive groups, showing higher mutation frequencies in the sensitive cohort. (right) Violin plot showing GSVA-derived HR pathway activity scores, which were significantly higher in the resistant group (P = 0.0316, Student's t-test).

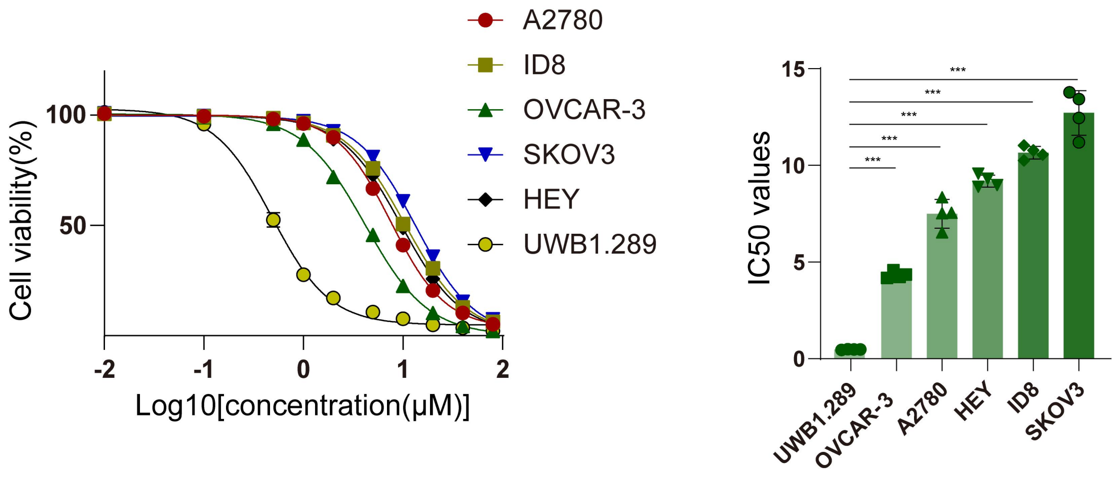

Figure s4. Differential sensitivity of ovarian cancer cell lines to anlotinib treatment. (Left) Dose–response curves showing cell viability of six ovarian cancer cell lines (A2780, OVCAR-3, SKOV3, HEY, ID8, and UWB1.289) following 48-hour exposure to increasing concentrations of anlotinib. (Right) Comparison of IC50 values derived from nonlinear regression analysis demonstrates that the HR-deficient UWB1.289 and OVCAR-3 cells were more sensitive to anlotinib than the HR-proficient lines (A2780, HEY, ID8, and SKOV3). Data are presented as mean ± SD from three independent experiments. ***P < 0.001.

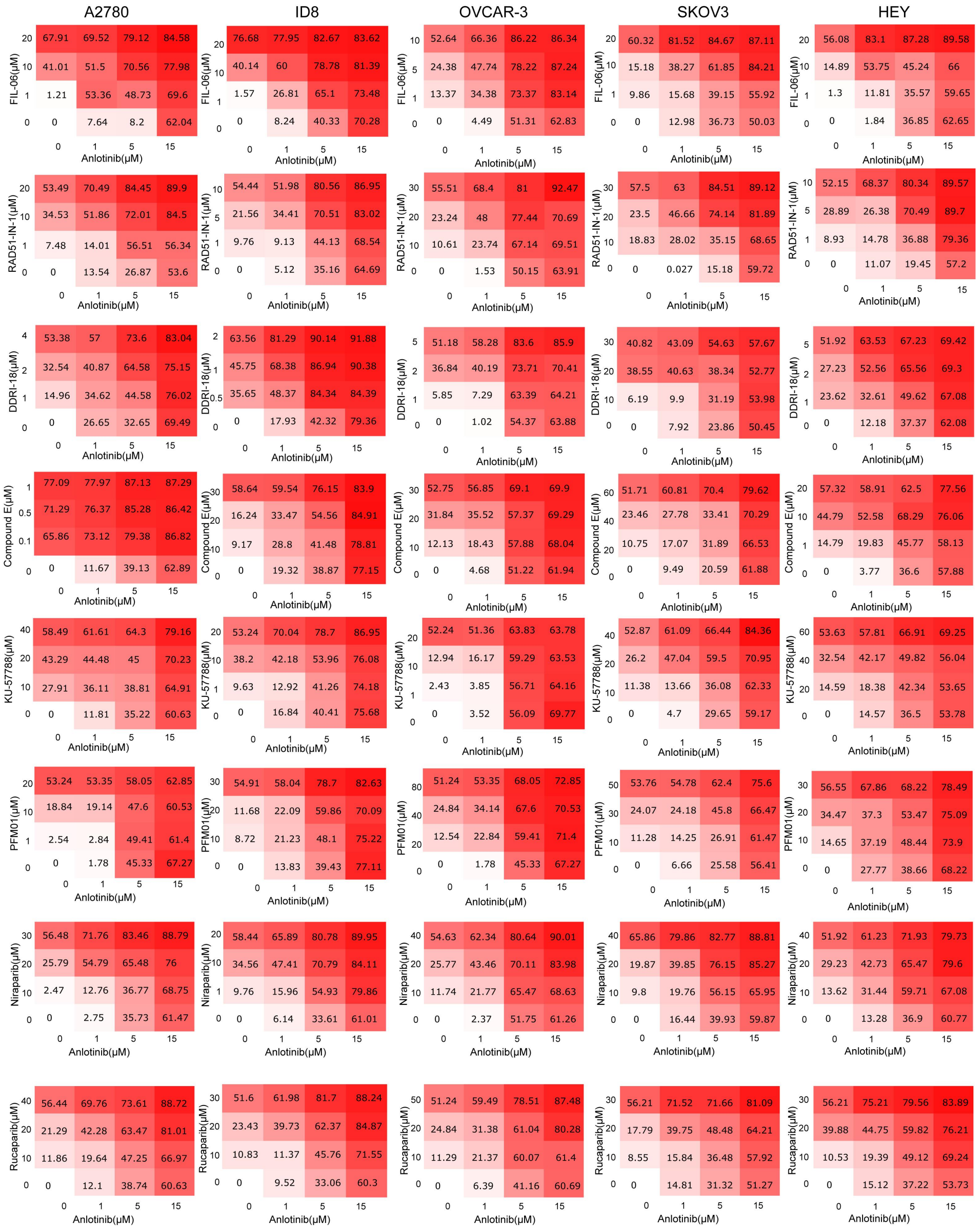

Figure s5. Representative dose–response heatmaps showing the effects of anlotinib combined with candidate pathway inhibitors across ovarian cancer cell lines.

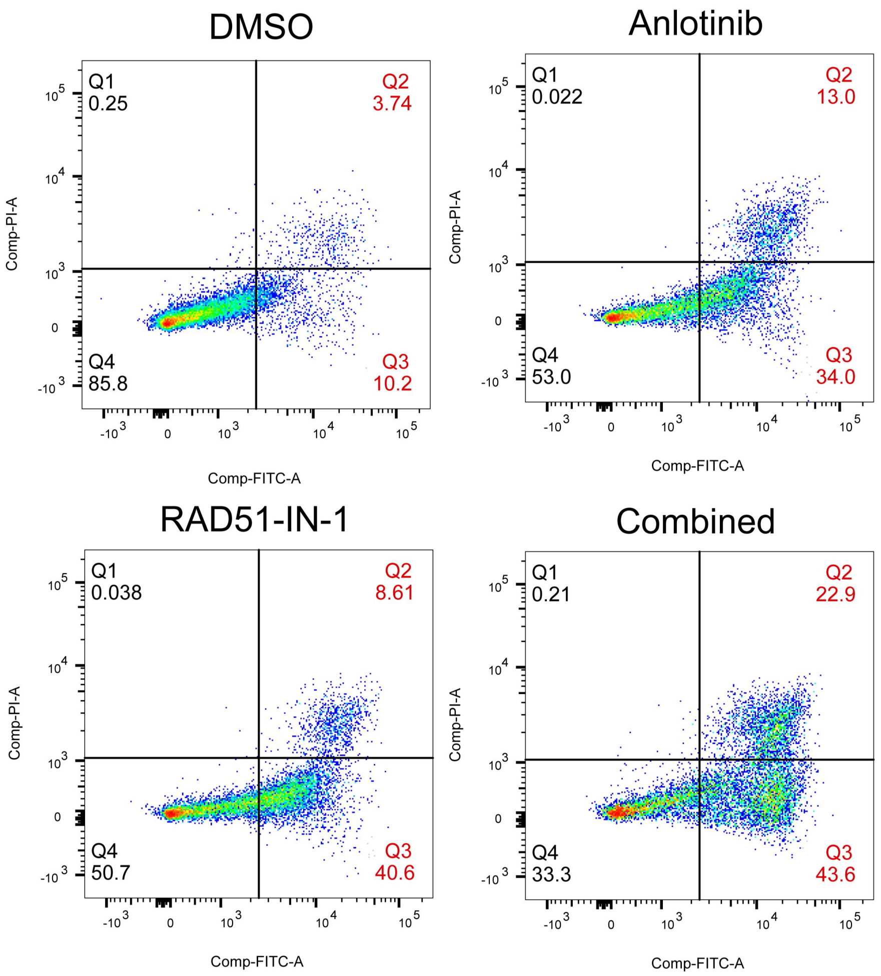

Figure s6. Flow cytometry analysis of apoptosis (annexin V–FITC/PI staining) in OVCAR-3 cells subjected to the indicated treatments. Compared with monotherapies, combined treatment markedly increased apoptotic cell death.

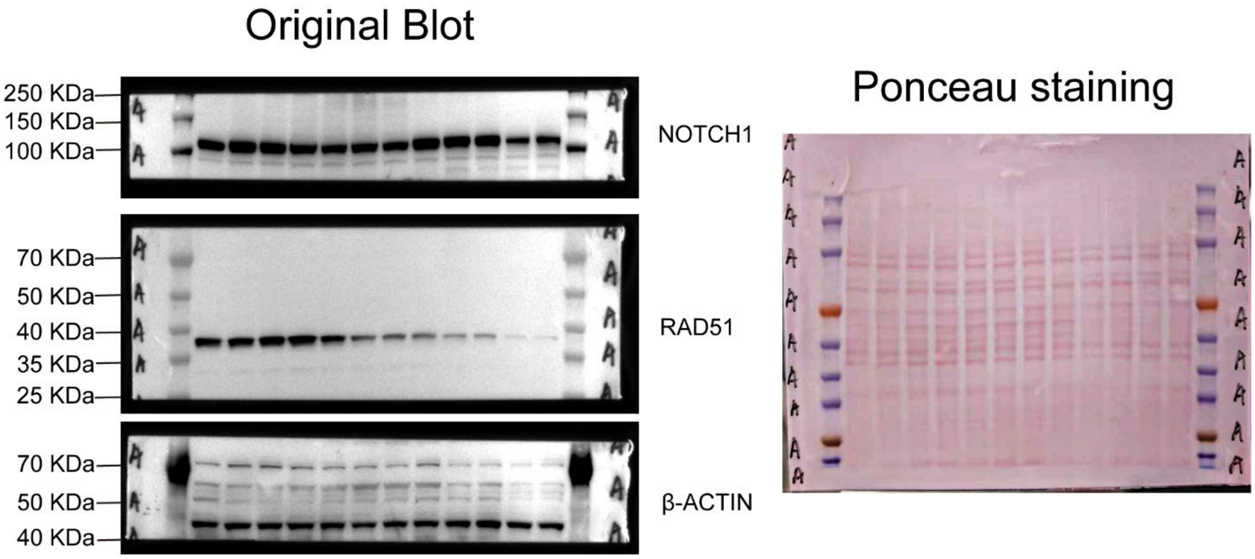

Figure s7. Ponceau S staining of the membrane following protein transfer, confirming uniform protein loading and efficient transfer across all samples.

Table s1. Comparison of mutation frequencies between anlotinib-resistant and -sensitive ovarian cancer samples

Gene	Resistant (n/N)	Sensitive (n/N)	Resistant (%)	Sensitive (%)	P value
ARID2	3/6	0/12	50.0%	0.0%	0.0245
NOTCH1	3/6	1/12	50.0%	8.3%	0.0833
PRKD1	3/6	1/12	50.0%	8.3%	0.0833
TBX3	3/6	1/12	50.0%	8.3%	0.0833
WNK2	3/6	1/12	50.0%	8.3%	0.0833
NCOR2	2/6	0/12	33.3%	0.0%	0.098
KMT2B	4/6	2/12	66.7%	16.7%	0.107
PDZD2	4/6	2/12	66.7%	16.7%	0.107
AFF3	0/6	5/12	0.0%	41.7%	0.114
FLG	0/6	5/12	0.0%	41.7%	0.114
GNAS	0/6	5/12	0.0%	41.7%	0.114
NUMA1	0/6	5/12	0.0%	41.7%	0.114
POLE	0/6	5/12	0.0%	41.7%	0.114
APC	2/6	1/12	33.3%	8.3%	0.245
ARID1A	2/6	1/12	33.3%	8.3%	0.245
ASXL1	0/6	4/12	0.0%	33.3%	0.245
FCRL4	0/6	4/12	0.0%	33.3%	0.245
AKAP9	3/6	2/12	50.0%	16.7%	0.268
ALK	3/6	2/12	50.0%	16.7%	0.268
BCOR	3/6	2/12	50.0%	16.7%	0.268
CREBBP	3/6	2/12	50.0%	16.7%	0.268
ERBB3	3/6	2/12	50.0%	16.7%	0.268
NIN	3/6	2/12	50.0%	16.7%	0.268
NOTCH2	3/6	2/12	50.0%	16.7%	0.268
NCOR1	1/6	6/12	16.7%	50.0%	0.316
POLD1	4/6	4/12	66.7%	33.3%	0.321
FAT1	3/6	3/12	50.0%	25.0%	0.344
PLCG1	3/6	3/12	50.0%	25.0%	0.344
ANKRD11	2/6	2/12	33.3%	16.7%	0.569
SIN3A	2/6	2/12	33.3%	16.7%	0.569

Values represent number and percentage of samples harboring mutations in each gene. *P* values were obtained using Fisher's exact test.

Table s2. Comparison of VEGFR related genes mutation frequencies between anlotinib-resistant and -sensitive groups

Gene	Resistant (n/N)	Sensitive (n/N)	Resistant %	Sensitive %	P value
**TIE1**	1/6	4/12	16.7 %	33.3 %	0.63
**NRP2**	1/6	3/12	16.7 %	25.0 %	1.00
**PIK3CD**	1/6	3/12	16.7 %	25.0 %	1.00
**FLT1**	1/6	2/12	16.7 %	16.7 %	1.00
**PIK3CG**	0/6	3/12	0 %	25.0 %	0.53
**VEGFA**	0/6	2/12	0 %	16.7 %	0.52
**VEGFC**	0/6	2/12	0 %	16.7 %	0.52
**FLT4**	0/6	2/12	0 %	16.7 %	0.52
**VEGFB**	0/6	1/12	0 %	8.3 %	1.00
**NRP1**	1/6	0/12	16.7 %	0 %	0.33
**VEGFD**	0/6	0/12	0 %	0 %	—
**PGF**	0/6	0/12	0 %	0 %	—
**KDR**	0/6	0/12	0 %	0 %	—

Values represent number and percentage of samples harboring mutations in each gene. *P* values were obtained using Fisher's exact test.

Table s3. Baseline characteristics of patients enrolled in the Bevacizumab cohort

Patient ID	Age (years)	FIGO Stage	BRCA Status	Recurrence	PFS (months)	Response
P164	53	IIIC	Wildtype	Yes	4	resistant
P169	62	IVB	Wildtype	Yes	5	resistant
P276	66	IIIC	Mutant	Yes	12	resistant
P309	55	IIIC	Wildtype	Yes	11	resistant
P311	52	IIIC	Wildtype	Yes	10	resistant
P314	61	IVB	Wildtype	Yes	7	resistant
P337	59	IVB	Wildtype	Yes	1	resistant
P343	68	IIIC	Wildtype	Yes	1	resistant
P394	48	IIIC	Mutant	Yes	10	resistant
P395	56	IIIC	Wildtype	Yes	10	resistant
P398	44	IIIC	Mutant	Yes	9	resistant
P414	70	ⅣB	Wildtype	Yes	9	resistant
P89	68	IIIC	Wildtype	Yes	6	resistant
P316	62	ⅢC	Mutant	Yes	14	sensitive
P326	51	IVB	Mutant	No	22	sensitive
P177	50	IIIC	Mutant	Yes	24	sensitive
P379	48	IVB	Mutant	No	17	sensitive
P134	50	IIIC	Wildtype	Yes	29	sensitive
P313	53	IIIC	Wildtype	Yes	14	sensitive
P302	61	IIIC	Mutant	Yes	14	sensitive

P307	60	IIIC	Wildtype	No	23	sensitive
P418	48	IIIC	Mutant	No	25	sensitive
P431	67	IVB	Wildtype	No	15	sensitive
P432	63	IIIC	Mutant	No	15	sensitive
P62	63	IIIC	Mutant	Yes	27	sensitive
P100	45	IIIC	Wildtype	Yes	23	sensitive
PM9	55	IVB	Mutant	No	28	sensitive

All patients included in this study had high-grade serous ovarian carcinoma (HGSOC). All BRCA mutations reported in this cohort were pathogenic germline mutations.

Data s1. Source data for selected figures.
